# Supplementary material for: Prevalence of Congenital Heart Disease and Associated Factors among Infants with Myelomeningocele: A cross-sectional study
Source: PLoS One. 2026 Jun 1;21(6):e0350419. doi: 10.1371/journal.pone.0350419 (PMC13225379; doi:10.1371/journal.pone.0350419)
Supplement: Attachment — Submitted filename: Response to reviewers.pdf [file pone.0350419.s004.pdf]

1. Is the manuscript technically sound, and do the data support the conclusions?

The manuscript must describe a technically sound piece of scientific research with data that supports the conclusions. Experiments must have been conducted rigorously, with appropriate controls, replication, and sample sizes. The conclusions must be drawn appropriately based on the data presented.

Reviewer #1: Partly
Reviewer #2: Yes
Response: We have revised the manuscript to clarify the study design, methodology, and how the data support the conclusions to ensure technical soundness.

2. Has the statistical analysis been performed appropriately and rigorously?

Reviewer #1: No
Reviewer #2: Yes
Response: We have updated the statistical analysis section, detailing methods, test selection, and inclusion of confidence intervals and p-values to ensure rigorous and transparent analysis.

3. Have the authors made all data underlying the findings in their manuscript fully available?

The PLOS Data policy requires authors to make all data underlying the findings described in their manuscript fully available without restriction, with rare exception (please refer to the Data Availability Statement in the manuscript PDF file). The data should be provided as part of the manuscript or its supporting information, or deposited to a public repository. For example, in addition to summary statistics, the data points behind means, medians and variance measures should be available. If there are restrictions on publicly sharing data—e.g. participant privacy or use of data from a third party—those must be specified.

Reviewer #1: Yes
Reviewer #2: No
Response: Due to the sensitive nature of pediatric patient data, the full dataset cannot be publicly shared; however, de-identified data are available upon reasonable request from the institutional ethics committee.

4. Is the manuscript presented in an intelligible fashion and written in standard English?

PLOS ONE does not copyedit accepted manuscripts, so the language in submitted articles must be clear, correct, and unambiguous. Any typographical or grammatical errors should be corrected at revision, so please note any specific errors here.

Reviewer #1: No
Reviewer #2: Yes
Response: We have carefully edited the manuscript to improve grammar, clarity, readability, and ensure it is written in standard English.

Reviewer 1

Reviewer Comment: Potential overfitting in the multivariable logistic regression model
The multivariable logistic regression analysis raises concerns regarding model stability. Only 19 cases of CHD were identified among the 265 infants included in the study. Conventional methodological recommendations suggest approximately 10 outcome events per predictor variable to ensure reliable estimates. Given the limited number of events, the inclusion of several predictors in the multivariable model may result in overfitting and unstable estimates. This is reflected in the relatively wide confidence intervals reported for several adjusted odds ratios. The authors should clarify the number of variables included in the final model and consider reducing the number of predictors or acknowledging this limitation more explicitly in the discussion.

Author Response: We thank the reviewer for highlighting this important concern. We included six variables in the final multivariable logistic regression model and acknowledge in the limitations section that, given only 19 CHD events, this may have resulted in overfitting and relatively wide confidence intervals. We have explicitly discussed this limitation in the revised manuscript to ensure transparency regarding model stability.

Reviewer Comment: The manuscript states that variables with $p < 0.05$ in bivariable analysis were included in the multivariable model. Selecting variables solely based on statistical significance in bivariable analysis is not generally recommended because it may exclude clinically relevant confounders and introduce bias in the final model. The authors should provide a clearer rationale for the model-building strategy and consider including variables based on clinical relevance or prior literature.

Author Response: We have clarified in the Bivariable Analysis section that independent variables were selected based on clinical relevance and prior literature, in addition to statistical significance, and were adjusted accordingly to ensure robust and unbiased estimates.

Reviewer Comment: The study included only infants with MMC who underwent echocardiographic evaluation. Infants who did not undergo echocardiography were excluded from the analysis. This approach may introduce selection bias when estimating the prevalence of CHD, particularly if echocardiography was performed selectively (for example, in infants with clinical suspicion of cardiac disease). The authors should clarify whether echocardiography was performed routinely in all infants with MMC or only when clinically indicated and discuss how this may affect the reported prevalence.

Author Response: Echocardiography was performed routinely for all infants with MMC. Ten patients were excluded because they disappeared before echocardiography and their caregivers could not be contacted by phone. We have clarified that echocardiography was done routinely in the Study Area, Period, and Design section of the Methods.

Reviewer Comment: The description of the study population is somewhat confusing. In the methods section, the total MMC population used for sample size correction is reported as N = 225, whereas the final analytical sample includes 265 infants. The relationship between these numbers should be clarified. It would be helpful to include a clearer description of the patient

selection process, ideally with a flow diagram showing the number of cases identified, excluded, and included in the final analysis.

Author Response: We appreciate the reviewer's observation. The mention of N = 225 in the manuscript was an error. The total number of infants with MMC in our hospital during the study period was 265. After recalculating the sample size with the correct population, the required sample size was smaller than the total available population. Since all 265 infants could be included, we analyzed the entire population. This has been clarified in the Methods section, and a flow diagram illustrating patient selection, exclusions, and inclusion in the final analysis has been provided.

Reviewer Comment: The authors excluded PFO and small PDA from the definition of CHD. Because definitions of CHD vary across studies, this choice should be more clearly justified. Excluding these lesions may lead to an underestimation of prevalence and may limit the comparability of the findings with previous studies. The authors should clarify which specific cardiac lesions were included in the CHD classification and discuss the implications of this definition.

Author Response: We thank the reviewer for this comment. Patent foramen ovale (PFO) and small patent ductus arteriosus (PDA) were excluded from the definition of CHD based on a review of previous studies, as most studies in the literature also exclude these lesions due to their high likelihood of spontaneous closure and minimal clinical significance. We have clarified in the Methods section, under the exclusion criteria, why these lesions were excluded.

Reviewer Comment: Internal inconsistency in the interpretation of prevalence
The results report a prevalence of CHD of 7.2%, yet the conclusion states that CHD affected "more than one in ten cases." This statement is inconsistent with the reported prevalence and should be corrected.

Author Response: We thank the reviewer for pointing this out. The conclusion has been corrected to accurately reflect the reported prevalence of CHD as 7.2%.

Reviewer Comment: Reporting of multicollinearity diagnostics
The methods section states that variance inflation factor (VIF) was used to assess multicollinearity, with a threshold of >10. However, the actual VIF values are not reported. The authors should provide these values or summarize the results of this assessment.

Author Response: We thank the reviewer for this comment. Maternal age and history of spontaneous abortion were the only variables with multicollinearity issues, and their VIF values have now been provided in the table 3.

Reviewer Comment: Missing cross-references in the manuscript
In the results section, several references appear as "Error! Reference source not found." These should be corrected before publication.

Author Response: We thank the reviewer for pointing this out. All missing cross-references have been corrected and verified throughout the manuscript.

Reviewer Comment: Title
The title could be streamlined to improve clarity and readability. Removing the detailed description of the recruitment center would make the title more concise while still preserving the key message of the study.

Author Response: We appreciate the reviewer's suggestion and have revised the title to make it more concise while retaining the essential focus of the study.

Reviewer Comment:
The manuscript would benefit from careful language editing to improve clarity and readability. Several grammatical and stylistic issues are present, including inconsistent punctuation and spacing around citations, unnecessary capitalization of variables, redundant phrasing, incorrect sentence structure, repetitive wording, and non-idiomatic expressions.

Author Response:
We thank the reviewer for highlighting these issues. The manuscript has been thoroughly revised to improve clarity and readability. Punctuation and spacing around citations have been corrected, capitalization of variables standardized, redundant phrasing removed, sentence structures simplified, repetitive wording minimized, and non-idiomatic expressions replaced with clearer alternatives (e.g., "in this cohort" instead of "in our cases"). These changes enhance the overall readability and consistency of the manuscript.

Reviewer 2

Reviewer Comment:
Page 10, last paragraph: the reference should be added properly instead of a computer error warning. The English should be revised to reflect a more academic style.

Author Response:
We have corrected the reference error in the last paragraph of the discussion so that the citation now appears correctly. Additionally, the paragraph has been revised for clarity and to reflect a more formal, academic writing style.

Reviewer comment: *"Given the considerable heterogeneity of congenital heart disease, a more detailed section specifying which types should be included, from low-risk conditions such as atrial septal defect (ASD) to complex anomalies such as hypoplastic left heart syndrome (HLHS), is warranted. The author should elaborate on this key point in the results section."*

Author response: "We thank the reviewer for this insightful comment. We have revised the results section to provide a detailed description of the CHD types observed, ranging from low-risk lesions such as atrial septal defect (ASD), patent ductus arteriosus (PDA), and perimembranous ventricular septal defect (PM VSD) to severe anomalies including complete atrioventricular septal defect (AVSD), tetralogy of Fallot (TOF), and hypoplastic left heart

syndrome (HLHS) (see Supplementary File 1). This revision clarifies the spectrum of CHDs in our cohort."

Reviewer comment:

"The influence of drug use, folate, alcohol, and similar factors should be discussed in both the results and discussion sections. Even if no major correlation with a single risk factor is observed at univariate analysis, the author should justify this and compare it with other reports."

Author response:

"We thank the reviewer for this important suggestion. We have revised the results and discussion sections to address the potential influence of maternal drug use, folate supplementation, and alcohol consumption. Although univariate analysis did not reveal significant associations with any single risk factor, we have provided an explanation and compared our findings with previous studies, highlighting the limitations due to small sample sizes."

Reviewer comment:

"Few papers are available in this region, which is one of the author's strong points. However, the paper does not clearly highlight this advantage, especially in the discussion section. Make sure to explicitly mention the lack of research in this region as a key contribution in the discussion."

Author response:

"We thank the reviewer for this insightful comment. We have revised the discussion section to emphasize that data on congenital heart disease (CHD) in infants with MMC are limited in this region. By presenting findings from our cohort, this study addresses a significant knowledge gap and contributes valuable regional data to the existing literature."
