# Supplementary material for: Prevalence of Congenital Heart Disease and Associated Factors among Infants with Myelomeningocele: A cross-sectional study
Source: PLoS One. 2026 Jun 1;21(6):e0350419. doi: 10.1371/journal.pone.0350419 (PMC13225379; doi:10.1371/journal.pone.0350419)
Supplement: Attachment — Submitted filename: REsponse_To_REviewers_auresp_2.pdf [file pone.0350419.s005.pdf]

Reviewer Comment:
Unresolved cross-references remain in the manuscript, including multiple instances of "Error! Reference source not found." in the Results section and the sample size paragraph of the Methods section. These placeholders indicate missing or improperly linked figures/tables, making parts of the manuscript difficult to interpret. All cross-references should be resolved before resubmission.

Response:
We sincerely thank the reviewer for this important observation. We acknowledge that the "Error! Reference source not found." placeholders appeared due to broken internal cross-references during manuscript formatting. This issue has now been fully corrected. All figures and tables have been properly re-linked to their corresponding in-text citations, and the manuscript has been carefully reviewed to ensure that all cross-references are correctly displayed. The revised version contains no unresolved references, and all Results and Methods sections are now fully readable and appropriately linked to the relevant figures and tables.

Reviewer Comment:
The Data Analysis section states that independent variables with $p < 0.05$ in bivariable analysis were included in the multivariable model, which is inconsistent with the stated approach of selecting variables based on clinical relevance, prior literature, and potential confounding. The manuscript should present a single coherent variable selection strategy.

Response:
We thank the reviewer for this important and insightful comment. We agree that the previous wording in the Data Analysis section was not fully consistent with our intended model-building strategy.

Accordingly, we have revised the Data Analysis section to clearly and consistently reflect that variables included in the multivariable logistic regression model were selected based on a combination of clinical relevance, evidence from prior literature, and consideration of potential confounding effects, in addition to bivariable analysis results ($p < 0.05$) as a supportive criterion.

Reviewer Comment:
The authors acknowledge that including six predictor variables with only 19 CHD events may have resulted in relatively wide confidence intervals. The reviewer suggests further contextualization by noting that penalised regression methods (e.g., Firth logistic regression) are specifically designed for sparse outcome data and may be preferable in future studies. This can be addressed with a single sentence in the limitations section without reanalysis.

Response:
We thank the reviewer for this valuable suggestion. We agree that further contextualization of the limitations related to sparse outcome data strengthens the methodological discussion. In response, we have added a sentence to the Limitations section acknowledging that penalised regression approaches, such as Firth logistic regression, are specifically designed to reduce bias and improve estimate stability in settings with few events and may be more appropriate for future studies with similar data structures.
